# The Definition of a Prolonged Intensive Care Unit Stay for Spontaneous Intracerebral Hemorrhage Patients: An Application with National Health Insurance Research Database

**DOI:** 10.1155/2014/891725

**Published:** 2014-07-14

**Authors:** Chien-Lung Chan, Hsien-Wei Ting, Hsin-Tsung Huang

**Affiliations:** ^1^Department of Information Management, Yuan Ze University, 135 Yuan-Tung Road, Chungli, Taoyuan 32003, Taiwan; ^2^Department of Neurosurgery, Taipei Hospital, Ministry of Health and Welfare, No. 127, Su-Yuan Road, Hsin-Chuang District, New Taipei City 24213, Taiwan; ^3^Medical Affairs Division, National Health Insurance Administration, Ministry of Health and Welfare, No. 140, Section 3, Hsin-Yi Road, Taipei 10634, Taiwan

## Abstract

*Introduction.* Length of stay (LOS) in the intensive care unit (ICU) of spontaneous intracerebral hemorrhage (sICH) patients is one of the most important issues. The disease severity, psychosocial factors, and institutional factors will influence the length of ICU stay. This study is used in the Taiwan National Health Insurance Research Database (NHIRD) to define the threshold of a prolonged ICU stay in sICH patients. *Methods.* This research collected the demographic data of sICH patients in the NHIRD from 2005 to 2009. The threshold of prolonged ICU stay was calculated using change point analysis. *Results.* There were 1599 sICH patients included. A prolonged ICU stay was defined as being equal to or longer than 10 days. There were 436 prolonged ICU stay cases and 1163 nonprolonged cases. *Conclusion.* This study showed that the threshold of a prolonged ICU stay is a good indicator of hospital utilization in ICH patients. Different hospitals have their own different care strategies that can be identified with a prolonged ICU stay. This indicator can be improved using quality control methods such as complications prevention and efficiency of ICU bed management. Patients' stay in ICUs and in hospitals will be shorter if integrated care systems are established.

## 1. Introduction 

Length of stay (LOS) in the intensive care unit (ICU) is one of the most important factors that influence health management. There are several factors that influence the ICU LOS: medical severity factors, psychosocial factors, and institutional factors [1]. Some studies have treated LOS as a hospital cost [2]. There are also many methods and management strategies that can influence the LOS. A higher nurse-patient ratio results in a lower hospital LOS [3]. The distributions of LOS analyzed by disease or institute are frequently skewed to the right. The mean, median, and range require more in-depth analysis for LOS studies [4]. However, the threshold of a prolonged LOS is useful for managers for analysis of the quality of care and hospital costs for diseases or institutes. The definition of a prolonged ICU stay varies by hospital type, ICU type, and also different diseases [5–10]. The severity of stroke, medical complications, and degree of disability are factors that influence the length of stay of patients [11–15].

Spontaneous intracerebral hemorrhage (sICH) is one of the most important diseases. The incidence of sICH varies by sex, age, and ethnic group [16–22]. The annual mortality rates of sICH vary in different countries but overall are around 50% [20, 23–25]. It represents around 10~35% of stroke patients and causes a higher mortality and morbidity than other strokes and costs much more in terms of medical facilities [16, 22, 26–29]. Koton et al. [30] constructed a prolonged length of stay score. They defined a prolonged LOS of stroke patients in acute wards as 7 days and suggested that the severity of stroke, comorbidities, prior disabilities, and sICH are all important factors affecting the length of stay in stroke patients. Luker et al. [15] also concluded that stroke severity, previous independence, comorbidities, day of admission, stroke unit admission, and LOS are outcome factors for stroke patients. Actually, sICH and LOS are part of the outcome evaluation factors. According to previous research, the quality of intensive care can be a good indicator of the outcome of sICH. However, few studies have discussed the definition of a prolonged ICU stay in sICH patients.

Most sICH patients will be admitted to ICU. However, the resources of ICUs are limited. These patients have one of the most important diseases and will utilize the ICU frequently. Therefore, evaluation of ICU resource allocation of sICH patients is very important. This study analyzed the Taiwan National Health Insurance Research Database (NHIRD) to define the threshold of a prolonged ICU stay in sICH patients. Other than the severity of the disease, the causes of a prolonged ICU stay arise from the management methods of hospitals or institutes. The result will be a good indicator of facilities allocation and outcome prediction for sICH patients.

## 2. Method

This research analyzed the Longitudinal Health Insurance Database (LHID2005) in the NHIRD from 2005 to 2009. A dataset of one million subjects was constructed from all subjects with National Health Insurance in 2005 by random sampling in Taiwan. The dataset used in this study contained outpatients and inpatients claims data with details recorded for each visit/stay, and the registry file for beneficiaries was processed to identify the demographic data from 2001 to 2009 [31]. Because the sampled one million subjects were patients in 2005, the subjects were definitely alive before 2005. It would severely increase the mortality rate if subjects prior to 2005 were included. Therefore, this study only used the NHIRD from 2005 to 2009 as the analysis base to prevent bias of mortality.

**Figure 1:**
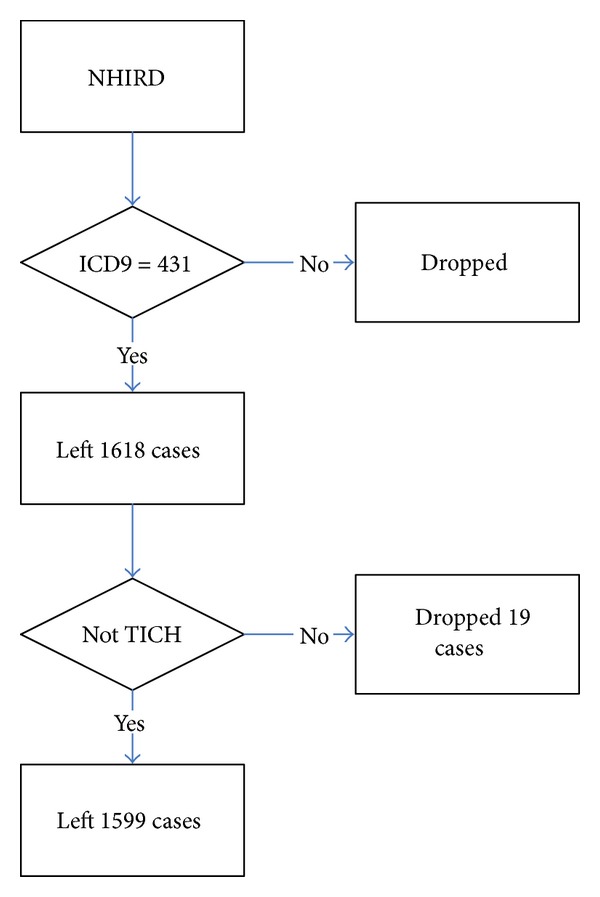
Flow chart of data management of the NHIRD.

The inclusion criteria of patients in this study were patients with a first attack of sICH whose diagnosis ICD9 code was 431. All patient data were collected at admission for a first sICH attack. There were a total of 1618 sICH cases included. Patients who were admitted due to traumatic intracranial hemorrhage (TICH) whose diagnosis ICD9 code was from 800 to 804.99, from 850 to 854.19, 959.01, and 959.09 were all excluded. There were 19 cases dropped owing to the exclusion criteria, and 1599 cases remained in total. The process of data management is shown in Figure 1. The admission cost, ICU admission and discharge dates, admission and discharge dates, and major admission comorbidities were all recorded. Multiple comorbidities were defined as patients who had 2 or more than 2 kinds of major admission comorbidities. Major admission comorbidities were defined as the first 5 major comorbidities collected in the NHIRD.

This study used change point analysis to find the threshold of a prolonged ICU stay in sICH patients. Change point analysis originated from studies of quality control [32, 33]. It can be used to solve problems of thresholds, identify the existence of any change point, and find the change point if there is one [34]. This method is used in many fields, including thresholds of ecological and biological processes, software reliability estimations, financial problems, changes of control charts and the status of manufacturing, finding trends and growth rate functions, flood season segmentation, and also medical problems [8, 35–38]. This study used the software combination of cumulative sum charts (CUSUM) and bootstrapping to detect the definition of the threshold for a prolonged ICU stay [39]. We usually construct a CUSUM chart and find the cumulative sum of the differences between individual data values and the mean. If there is no shift in the mean of the data, the chart will be relatively flat with no pronounced changes in slope. The range will also be small. A dataset with a shift in the mean will have a slope change at the data point at which the change occurred, and the range will be relatively large [40, 41].

After the threshold of a prolonged ICU stay was defined, the percentages of prolonged ICU stays for sICH patients in hospitals with different training capacities, those with different ownerships, and those in different regions were also compared. Training hospitals were divided into medical centers, regional hospitals, and local hospitals. Medical centers performed the most staff training. Local hospitals provide medical care for the local area and do not have a great training burden. Hospital ownership was divided into government hospitals, public medical school hospitals, military hospitals, veterans hospitals, religious hospitals, private medical school hospitals, and private hospitals. There were 6 divisions: the Taipei division, Northern division, Central division, Southern division, Kaoping division, and Eastern division. The Taipei division is the area of the capital of Taiwan. The Eastern division is a rural area in Taiwan.

Student's *t*-test was used for the continuous data. The *χ* ^2^ test was used for the categorical data. Kaplan-Meier survival analysis was used for the survival analysis of sICH patients. Student's *t*-test, *χ* ^2^ test, and Kaplan-Meier survival analysis were calculated using SPSS version 12.0 (SPSS Inc., Chicago, IL, USA). The change points were analyzed using Change-Point Analyzer version 2.3 (Taylor Enterprises, Inc., Libertyville, IL, USA). Statistical significance was defined as *P* < 0.05.

## 3. Results

**Figure 2:**
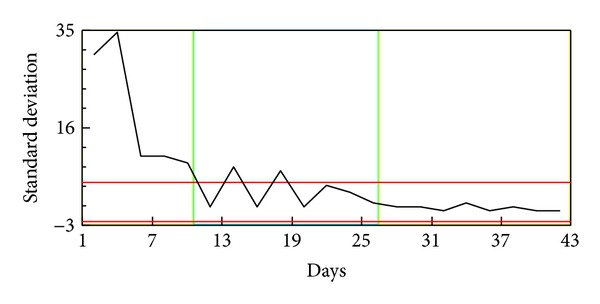
Plot of days against standard deviation.

**Figure 3:**
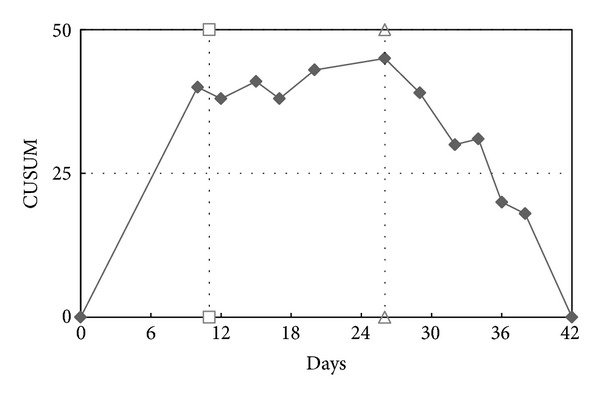
CUSUM (cumulative sum control) chart of days standard deviation.

**Figure 4:**
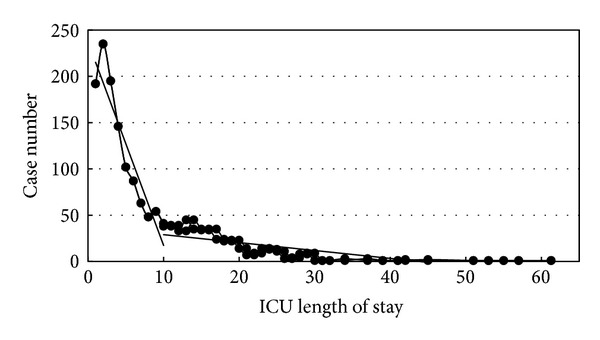
Case distribution of ICU days and change point analysis results.

With the cumulative sum control chart (CUSUM chart) of days standard deviation, standard deviation changes were found for the 11th day and the 23rd day (Figures 2 and 3 and Table 1). A prolonged ICU stay of sICH patients was defined as more than 10 days (Figure 4). According to this definition, 436 cases (27.3%) had a prolonged ICU stay and the remaining 1163 cases (72.7%) did not have a prolonged ICU stay. There are 36.5% of the patients who were female. There was no significant difference in the gender ratio between the prolonged and nonprolonged ICU stay patients. The mean age of the sICH patients was 62.8 years (SD = 15.0). The prolonged ICU stay patients (64.7 years, SD = 14.0) were significantly older than the nonprolonged ICU stay patients (62.1 years, SD = 15.3) (*P* < 0.01). The mean admission LOS and ICU stay were 16.7 (SD = 12.0) and 7.8 (SD = 7.7), respectively. Both the hospital LOS (27.1 days, SD = 11.1) and the ICU stay (18.2 days, SD = 7.2) of the prolonged ICU stay patients were longer than those of the nonprolonged ICU stay patients (13.1 days, SD = 10.3 and 3.9 days; SD = 2.5) (*P* < 0.001). The surgical intervention rate for the sICH patients was 25.5%, and the rate for the prolonged ICU stay patients (67.6%) was higher than that for the nonprolonged ICU stay patients (25.3%). Although the patient-day cost ratio of the nonprolonged ICU stay patients (56.4%) was higher than that of the prolonged ICU stay patients (43.6%) (*P* < 0.001), the ICU patient-day cost ratio of the prolonged ICU stay patients (63.3%) was higher than that of the nonprolonged ICU stay patients (36.7%) (Table 2).

The hospital cost of the prolonged ICU stay cases (US$11,036, SD = 4808) was significantly higher than that of the nonprolonged ICU stay cases (US$3,155, SD = 2510) (*P* < 0.001). If the total hospital fees cost is discussed, the prolonged ICU stay patients represented only 436 cases (27.3%) but cost more than half (56.7%) of the sICH patients' hospital care. The highest fees cost is the room fee, which represents around 34.5%, followed by medications fees (14.3%), surgical fees (13.1%), diagnostic fees (8.5%), doctor care fees (5.4%), and other treatment fees (24.2%). The percentage of diagnostic fees of the nonprolonged ICU stay cases (10.6%) was higher than that of the prolonged ICU stay cases (7.0%) (*P* < 0.01). The percentage of medication fees of the nonprolonged ICU stay cases (12.6%) was lower than that of the prolonged ICU stay cases (15.6%) (*P* < 0.05). There were no significant differences in the percentages of the other types of cost between the prolonged and nonprolonged ICU stay patients (Table 2).

**Figure 5:**
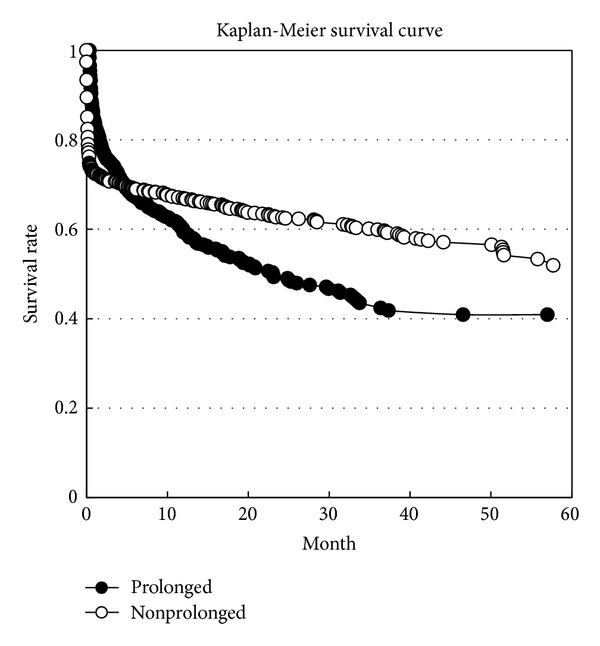
Survival rate curves of nonprolonged ICU stay patients and prolonged ICU stay patients (*P* < 0.001). The mortality rates of the patients with a prolonged/nonprolonged ICU stay were 50.7%/38.4%, respectively. The overall mortality rate was 41.8%.

The mean survival time (months) of the sICH patients was 36.0 months (95% CI = 34.5~37.5). That of the nonprolonged ICU stay patients (38.0, 95% CI = 36.2~39.7) was longer than that of the prolonged ICU stay patients (31.0, 95% CI = 28.2~33.7) (*P* < 0.01). The total mortality rate of the sICH patients was 41.8%. The mortality rate of the prolonged ICU stay cases (50.7%) was significantly higher than that of the nonprolonged ICU stay cases (38.4%), with the odds ratio being 1.674 (95% CI = 1.342~2.087) (*P* < 0.001). According to Figure 3, the patients with a nonprolonged ICU stay will die very quickly in the start time and slow down after surviving for more than one month. The survival lines of the prolonged ICU stay and nonprolonged ICU stay patients crossed at the 5th month. Finally, the patients with a nonprolonged ICU stay had a higher survival rate than those with a prolonged ICU stay (Figure 5).

This study also studied the major admission comorbidities of sICH patients. 30.6% of the sICH patients had multiple comorbidities. The patients with a prolonged ICU stay (39.2%) had a higher percentage of multiple comorbidities than the patients with a nonprolonged ICU stay (27.3%), with the odds ratio being 1.715 (95% CI = 1.360~2.161) (*P* < 0.001). The most common comorbidity of sICH patients was found to be hypertension (62.0%). The patients with a prolonged ICU stay (54.1%) had a lower percentage of having hypertension than the patients with a nonprolonged ICU stay (65.0%), with the odds ratio being 0.635 (95% CI = 0.508~0.794) (*P* < 0.001). The subsequent most common diseases were pulmonary diseases (18.2%). The patients with a prolonged ICU stay (37.6%) were at higher risk of having pulmonary diseases than the patients with a nonprolonged ICU stay (10.9%), with the odds ratio being 4.918 (95% CI = 3.764~6.426). 17.3% sICH patients had diabetes mellitus (DM). There was no significant difference in the percentage of DM between sICH patients with a prolonged and a nonprolonged ICU stay. There were 15.1% sICH patients with hydrocephalus. A higher percentage of patients with a prolonged ICU stay (28.9%) had hydrocephalus than patients with a nonprolonged ICU stay (10.0%), with the odds ratio being 3.699 (95% CI = 2.792~4.902). The incidences of the other common comorbidities in sICH patients were an old CVA (5.6%), heart diseases (3.8%), renal diseases (3.4%), liver diseases (2.6%), peptic ulcer (1.8%), and dementia (1.3%). There were no significant differences in the percentages of sICH patients with a prolonged and nonprolonged ICU stay with these comorbidities (Table 3).

This study evaluated the differences between hospitals of different classifications, including training capacity, ownership of the hospital, and region of the hospital. There were no significant differences in the female ratio and mortality within 30 days between hospitals, no matter what the classification. According to the classifications of training hospitals, most ICH patients stay in regional hospitals (881 cases); the rest stay in medical centers (571 cases) and local hospitals (147 cases). There were no significant differences in the surgical intervention ratio, mortality ratio, and prolonged ICU stay ratio among medical centers, regional hospitals, and local hospitals. However, the mean age of patients of medical centers (61.4 years, SD = 15.3) was significantly younger than those of regional (63.3 years, SD = 14.8) and local hospitals (64.8 years, SD = 14.3) (*P* < 0.05). Local hospitals (9.3 days, SD = 9.6) had the longest mean ICU days and regional hospitals (7.4 days, SD = 7.1) had the shortest mean ICU days (*P* < 0.05). Medical centers (US$6327, SD = 5582) had the highest hospital expenditure as compared with regional (US$4,701, SD = 4161) and local hospitals (US$4,942, SD = 4,137) (*P* < 0.001) (Table 4).

According to hospital ownership, most patients stay in private hospitals: private hospitals (1018 cases), private medical school hospitals (142 cases), and religious hospitals (56 cases). There were no significant differences in the mean age and mortality rates between hospitals of different ownerships. Private hospitals had a significantly lower ratio of prolonged ICU stays than public hospitals (*P* < 0.001). The veterans hospitals had the highest hospital expenditure (US$8,979, SD = 7,698) as compared with the other hospitals, and the rest are military hospitals (US$6,629, SD = 6,040). The religious hospitals (US$4,245, SD = 3,755) had the lowest hospital expenditure of all the hospitals (*P* < 0.001). The surgical intervention ratios of the veterans hospitals (50.0%) were the highest among other hospitals, and the public medical school hospitals (19.7%) had the lowest ratio of surgical intervention (*P* < 0.001). Both military hospitals (20.0 days, SD = 13.2 and 9.5 days; SD = 9.1) and veterans hospitals (21.7 days, SD = 14.1 and 11.8 days; SD = 11.2) had the highest hospital admission days and ICU days (*P* < 0.001) (Table 4).

According to the regions of hospitals, more than 1/4 of all the cases were in the Taipei division (409 cases). There were no significantly different ratios of surgical interventions and mortality rates among the divisions. The patients in the Taipei division had a higher ratio of a prolonged ICU stay than the patients in the other divisions (*P* < 0.01). The hospital admission days (20.6 days, SD = 14.2) and ICU days (9.0 days, SD = 9.2) of the Taipei division were both higher than those of the other divisions (*P* < 0.001). The Taipei division (US$6,646, SD = 5,940) and Northern division (US$5,522, SD = 4,527) had the highest hospital expenditures as compared with the other divisions (*P* < 0.001) (Table 4).

## 4. Discussion 

The Taiwan National Health Research Institute (NHRI) constructed the NHIRD, a nationwide research database, for medical research purposes [31]. Many researchers have used the NHIRD to study medical and epidemiological issues [42, 43]. Some researchers have also completed medical cost-related research using the NHIRD [42, 44]. The Cochrane systemic review found that some studies have treated LOS as a financial factor, which can be considered a surrogate for hospital cost [2]. The hospital LOS and the ICU stay will vary under different care policies in different countries and hospitals [1]. The care policies for sICH patients in different countries or regions differ, and ICH patients receive a high quality of care and incur a lower cost as they are covered by the Taiwan National Health Insurance [22]. A prolonged length of stay is defined as 7 days for stroke patients. One type of stroke patients is those who are classified as sICH patients [30]; however, there is no definition for sICH patients. Actually, sICH patients have a more severe illness, and these patients will have a longer ICU stay and will incur a greater hospital expenditure. This study found the definition of a prolonged ICU stay using the national health insurance database of Taiwan. This definition can be a good indicator for hospital management and resources evaluation for sICH patients.

Another benefit of the national health database is that it provides the opportunity for research when some randomized control trial studies are impossible, for example, the design of beneficial evaluation studies for surgical intervention. According to the knowledge and training of the neurosurgeon and the ethical implications, decision-making regarding surgical intervention is focused on the best interests of the patients [45]. We cannot design a “randomized control” study for surgical intervention studies. Large dataset research may solve these problems. Even though there can be no truly double/triple blind randomized control trial for surgical intervention studies, the results of NHIRD data mining may still reflect the reality of sICH patients' care.

Change point analysis is a group of methods used to solve problems of change [32, 33]. These methods solve the problems of thresholds, identify the existence of any change point, and find the change point if there is one [34]. Huang et al. used change point analysis and defined 16 days as a surgical ICU prolonged stay [8]. This study used the NHIRD and defined 10 days as the threshold of a prolonged ICU stay for sICH patients using change point analysis. The result is reliable for sICH patients because this database represents the national medical care status in Taiwan, and change point analysis did find a valuable threshold for a prolonged ICU stay.

Although they represent only around 27% of patients, ICH patients with a prolonged ICU stay spend almost half of total hospital patient-day and more than 60% of total ICU patient-day for the care facilities. The hospital admission cost of the patients with a prolonged ICU stay (US$11,036) was almost 3 times that of patients with a nonprolonged ICU stay (US$3,155). A prolonged ICU stay may represent both hospital cost and mortality indicators for sICH patients. According to Figure 5, we found that more than 25% of ICU patients with a nonprolonged ICU stay died within one month, and then their mortality rate decreased gradually. This group (the nonprolonged group) consisted of two types of patients: the first is patients with an illness too severe to survive (early mortality patients); the other is patients who have a real nonprolonged ICU stay. There is no doubt that this group will cost less in terms of medical facilities than prolonged ICU stay patients. The early mortality patients will cost less than those with a prolonged ICU stay due to less ICU occupation duration or death during ICU admission. The other patients, the real nonprolonged ICU stay patients, cost less in terms of ICU facilities than patients with a prolonged ICU stay. Both types of patient cost less in terms of medical facilities than those patients with a prolonged ICU stay.

Comorbidities influence not only the outcome but also the hospital admission LOS of patients [15, 46]. Some studies have mentioned that different individual comorbidities could influence the outcome of sICH patients. For example, some studies have used hypertension or DM as outcome indicators, and other researches found that respiratory failure or pneumonia influence the LOS and mortality rate of sICH patients [14, 29, 47, 48]. However, few studies have discussed the influence of multiple comorbidities in sICH patients. This study defined multiple comorbidities as patients who have 2 or more than 2 kinds of major admission comorbidities. Only 4 major comorbidities are recorded during admission in the NHIRD. Actually, the number of major comorbidities plays an important role in outcome measurement and influences the LOS of sICH patients. This study found that patients with multiple major comorbidities will have a poorer outcome and a prolonged LOS. These patients utilize more hospital resources than those with a single comorbidity or no comorbidities, no matter whether the comorbidities arise from previous disease history or complications. Although the previous disease history of patients cannot be changed, decreasing complications using quality control methods will lead to a better outcome and a shorter LOS for sICH patients [14].

This study found that the northern area of Taiwan had higher ICU facilities usage and medical expenditure for the care of ICH patients. In contrast, the Eastern division, which is in a rural area, had both a lower ratio of prolonged ICU stay and a lower medical expenditure for ICH patients. The different training capacities also influence the medical expenditure and the ratio of a prolonged ICU stay in ICH patients. Medical centers have the highest ratio of a prolonged ICU stay because more severe patients will be transferred to medical centers. It is interesting, though, that local hospitals have a high ratio too. Families do not need to care for patients when they are admitted to the ICU. Psychosocial factors influence the ratio of a prolonged ICU stay in local hospitals. This study also found that private hospitals have a lower ratio of a prolonged ICU stay and incur a lower medical expenditure than public hospitals. It might be concluded that private hospitals are more efficient in terms of patient care than public hospitals. These results also proved that the threshold of a prolonged ICU stay is a good indicator of hospitals utility in some diseases. Different hospitals have their own different care strategies for those with a prolonged ICU stay.

There are many factors that influence both mortality and ICU stay, for example, diseases severity, psychosocial problems, less integrated care, and institutional factors [1]. Due to the disabilities of patients, the most important issue for ICH patients is care after the disease has been stabilized. Research has shown that early mobilization will decrease the LOS in ICU [49]. Proactive palliative care consultations for high-risk patients, for example, ICH patients, will significantly decrease the LOS in ICU [50]. Decision support systems for ICU staff are also good tools for decreasing the LOS in ICU [51]. The integration of care after discharge from ICU is also an important issue related to a decreased LOS in the ICU. Patients stay in ICUs and hospitals for a shorter period of time if integrated care systems have been established, and these methods will reduce the medical expenditure paid by insurance systems [52]. This study also found that private hospitals have lower prolonged ICU stay rates than public hospitals. It can be concluded that efficiency in terms of hospital management can also decrease the LOS in ICU. Further evaluations are needed to identify methods that can decrease the LOS in ICU for sICH patients.

There were some limitations in this research. First, the NHIRD is an administrative database used for health insurance. Therefore, this database did not collect information regarding the site and depth of the ICH. Some secondary outcomes, such as disabilities and sequelae after sICH, were not recorded in the NHIRD. However, according to information technologies and data mining methods, we were still able to find some hidden facts and knowledge from the medical database used. Although they are retrospective data, such results or knowledge has been proven following further study to be good models for medical care [53–56]. Second, this study presented a general definition of a prolonged ICU stay for ICH patients. Patients will tend to have a prolonged ICU stay if they have more comorbidities/complications. Different comorbidities/complications or diseases have different influences on the ICU length of stay. New prolonged ICU stay definitions taking into account complications and comorbidities will be identified in future studies.

## 5. Conclusion

This study defined 10 days as a prolonged ICU stay using change point analysis. This study also showed that the threshold of a prolonged ICU stay is a good indicator of hospitals utilization in ICH patients. Different hospitals have their own different care strategies, which can be identified from a prolonged ICU stay. This definition can be a good indicator of quality control in hospital management. According to governmental strategies or health policies, the ICU and hospital lengths of stay will be shorter if integrated care systems are established. This could also reduce the medical cost paid by healthcare insurance systems.

## Figures and Tables

**Table 1:** Significant change points of days standard deviations.

Row	Confidence interval	Confidence level	From	To
11	(11, 15)	94%	15.725	3.6693
23	(23, 31)	94%	3.6693	0.52418

Confidence level for candidate changes = 50%; confidence level for inclusion in table = 90%; confidence interval = 95%; bootstraps = 1000.

**Table 2:** Demographic data of sICH patients from 2005 to 2009.

	Nonprolonged ICU stay (SD)	Prolonged ICU stay (SD)	Total (SD)
Case number (*n*)	1163	436	1599
Female case percentage	36.8%	35.6%	36.5%
Mean age∗∗	62.1 (15.3)	64.7 (14.0)	62.8 (15.0)
Mean admission days∗∗∗	13.1 (10.3)	27.1 (11.1)	16.7 (12.0)
Mean ICU days∗∗∗	3.9 (2.5)	18.2 (7.2)	7.8 (7.7)
Surgical intervention ratio∗∗∗	25.5%	67.7%	37.0%
Patient-day spend ratio∗∗∗	56.4%	43.6%	100%
ICU patient-day cost ratio∗∗∗	36.7%	63.3%	100%
Total cost percentage	43.3%	56.7%	100%
^†^Total hospital fees∗∗∗	3,155 (2510)	11,036 (4,808)	5304 (4,817)
Hospital room fees ratio	34.2%	34.7%	34.5%
Medications fees ratio∗	12.6%	15.6%	14.3%
Surgical fees ratio	13.7%	12.6%	13.1%
Diagnostic fees ratio∗∗	10.4%	7.0%	8.5%
Doctor care fees ratio	6.4%	4.7%	5.4%

*P < 0.05, **P < 0.01, and ***P < 0.001; ^†^unit is US dollars.

**Table 3:** Comorbidity influence of patients with a prolonged ICU stay and those with a nonprolonged ICU stay.

	Nonprolonged (*n* = 1163)	Prolonged (*n* = 436)	All (*n* = 1599)	Odds ratio
Multiple comorbidities∗∗∗	27.3%	39.2%	30.6%	1.715 (1.360~2.161)
Hypertension∗∗∗	65.0%	54.1%	62.0%	0.635 (0.508~0.794)
Pulmonary diseases∗∗∗	10.9%	37.6%	18.2%	4.918 (3.764~6.426)
Diabetes mellitus	16.9%	18.3%	17.3%	NP
Hydrocephalus∗∗∗	10.0%	28.9%	15.1%	3.669 (2.766~4.865)
Old CVA	5.3%	6.4%	5.6%	NP
Heart diseases	4.3%	2.5%	3.8%	NP
Renal disease	2.9%	4.8%	3.4%	NP
Liver disease	3.0%	1.6%	2.6%	NP
Peptic ulcer	1.8%	1.8%	1.8%	NP
Dementia	1.5%	0.7%	1.3%	NP

*P < 0.05, **P < 0.01, and ***P < 0.001.

DM: diabetes mellitus; CVA: old CVA that was not sICH; PU: peptic ulcer.

**Table 4:** Prolonged ICU stay ratios and other profile comparisons in different types of hospitals.

	Case number (*n*)	Surgical intervention ratio	Mortality within 30 days	Prolonged ICU stay ratio	^†^Total hospital fees
All cases	1599	37.00%	25.10%	27.30%	5304 (4817)
Training hospital (*P* value)		n.s.	n.s.	n.s.	0.000
Medical center	571	41.30%	25.20%	29.10%	6327 (5582)
Regional hospital	881	34.50%	24.00%	25.40%	4701 (4161)
Local hospital	147	34.70%	32.00%	31.30%	4942 (4137)
Hospital ownership (*P* value)		0.006	n.s.	0.002	0.000
Government hospital	180	30.60%	26.70%	24.40%	4772 (4379)
Public medical school hospital	61	19.70%	27.90%	31.10%	5213 (4160)
Military hospital	66	34.80%	19.70%	36.40%	6629 (6040)
Veterans hospital	76	50.00%	23.70%	46.10%	8979 (7698)
Religious hospital	56	37.50%	21.40%	21.40%	4245 (3755)
Private medical school hospital	142	41.50%	23.90%	29.60%	5543 (4558)
Private hospital	1018	37.60%	25.50%	25.50%	5068 (4518)
Hospital regions (*P* value)		n.s.	n.s.	0.005	0.000
Taipei division	409	36.20%	23.00%	32.00%	6466 (5940)
Northern division	212	42.50%	29.70%	27.80%	5522 (4527)
Central division	369	39.00%	21.70%	29.50%	5346 (4498)
Southern division	258	32.60%	26.70%	20.50%	4117 (3679)
Kaoping division	289	37.40%	26.00%	26.00%	4678 (4189)
Eastern division	62	27.40%	33.90%	14.50%	4495 (4605)

n.s.: nonsignificant; *P < 0.05, **P < 0.01, and ***P < 0.001; ^†^unit is US dollars.
